# Household and health system cost of malaria in sub Saharan-Africa: a systematic review

**DOI:** 10.1186/s12936-026-06060-2

**Published:** 2026-08-03

**Authors:** Yirgalem Shibiru Baruda, Yihalem Abebe Belay, Lelisa Fekadu Assebe

**Affiliations:** 1 Success Healthecon Consultancy PLC, Addis Ababa, Ethiopia; 2 Department of Public Health, College of Medicine and Health Sciences, Debre Markos University, Debre Markos, Ethiopia; 3 AHEFTA Consulting, Addis Ababa, Ethiopia

**Keywords:** Cost, Health system, Household, Malaria, Sub-Saharan Africa, Review

## Abstract

**Background:**

Malaria is a major public health problem in Sub-Saharan Africa (SSA), causing a considerable burden on households and health systems. This systematic review analyses the malaria treatment cost faced by households and health systems in Sub-Saharan Africa (SSA).

**Methods:**

The review was conducted according to the Preferred Reporting Items for Systematic Reviews (PRISMA) guideline. Studies reported malaria treatment costs incurred by households and the health system in SSA were included in the review. A pooled estimate for household and health system costs of malaria was conducted using the random effects model of the Residual Maximum Likelihood (REML) method. The analysis is conducted in STATA version 16. A total of 39 studies were included in the review, of which approximately 56% reported household costs and 23% reported health system costs.

**Results:**

The pooled estimated household cost per outpatient malaria episode was $12.55 (95% CI $9.58, $16.45, *I*^2^ = 96.29%, P < 0.01), while the pooled inpatient cost per severe malaria episode was $53.71 (95% CI $41.00, $70.31, *I*^2^ = 96.22%, P < 0.01). Households in the poorest quintile incurred the highest malaria treatment costs compared with those in the richest quintile. The pooled estimated health system cost per outpatient malaria episode was $19.91 (95% CI $10.77, $36.81, *I*^*2*^ = 92.44%, P < 0.01), while the health system inpatient cost per severe malaria was $64.03 (95% CI $48.86, $83.90,* I*^*2*^ = 93.01%, P < 0.01).

**Conclusions:**

Malaria imposed a substantial economic burden on health system and households in low-income countries in SSA. Our findings provide evidence to guide malaria programs planning and enhance resource allocation in SSA.

*PROSPERO registration number*: CRD420251266073.

**Supplementary Information:**

The online version contains supplementary material available at https://doi.org/10.1186/s12936-026-06060-2.

## Introduction

Malaria continuous to cause a high burden of illness and deaths in Low-Income Countries (LICs) [1]. It remains as a significant global health concern with substantial impacts on human health, social well-being, and economic development, according to the World Health Organization. Globally, 263 million malaria cases and 597,000 related deaths were reported in 2023 [2]. Nearly half of all cases worldwide were reported from Nigeria (30.9%), the Democratic Republic of the Congo (11.3%), Niger (5.9%) and Tanzania (4.3%) [2]. According to the US Centers for Disease Control and Prevention, malaria disproportionately affects vulnerable populations, including under five children and pregnant women. The disease not only leads to acute illness and mortality but also contributes to long-term health complications and disabilities. Malaria-related anaemia, cognitive impairment in children, and adverse pregnancy outcomes are some of the consequences that hinder individual and societal well-being [3].

The economic impact of malaria on households in SSA is profound and far-reaching. Malaria-related cost imposes a major financial burden on individuals and households [4]. The household costs of seeking malaria care, including consultations, diagnostic tests, and antimalarial medications, push families into poverty [5]. For instance, household malaria treatment costs contribute to the rise in households living in poverty by 2–3% in Ethiopia [6]. Thus, the burden of malaria in the region extends beyond high treatment costs, affecting economic conditions of the households [7].

Moreover, malaria treatment has a considerable economic burden on health systems, through direct medical expenses like medication, diagnostics, personnel and hospitalization, in the endemic areas [8]. In endemic areas such as Nigeria, these expenses consume up to 25% of health facilities budget [9]. Overall, the cost of treating malaria places a major strain on the health system in SSA, in particular in LICs [10].

Therefore, addressing the economic impact of malaria on both households and health systems requires comprehensive strategies to strengthen health systems and improve the affordability and accessibility of malaria prevention and treatment [11]. Accessible and affordable malaria diagnosis and treatment services are crucial to ensure prompt and appropriate care so as to reduce the economic burden of malaria to the household and health system.

The previous reviews have largely summarized malaria treatment costs in Sub-Saharan Africa by reporting ranges of minimum and maximum costs from household, health system, and societal perspectives [10, 12]. While useful for describing variability, these studies do not provide pooled cost estimates that can offer a more robust and comparable measure of the economic burden across settings [8]. In addition, evidence on aggregated household and health system costs remains limited, making it difficult for policymakers to fully understand the overall economic impact of malaria and to compare costs across contexts. Therefore, this systematic review aims to assess the pooled medical costs incurred by households and the health system for malaria treatment in SSA, thereby strengthening evidence for health policy, program planning, and resource allocation to reduce the economic burden of malaria in the region.

## Methods

### Search strategies and data sources

A comprehensive search was conducted in the following electronic databases: PubMed, Embase, Web of Science, and Google Scholar. Using the PECO framework (Population, Exposure, Comparator, and Outcomes), both simple and advanced searches were conducted in each of the databases using a combination of keywords related to malaria, patient costs, economic burden and SSA, as well as Medical Subject Headings (MeSH) terms. The search terms were adapted according to the specific requirements of each database by incorporating appropriate Boolean operators (AND, OR) and truncation symbols.

**Fig. 1:**
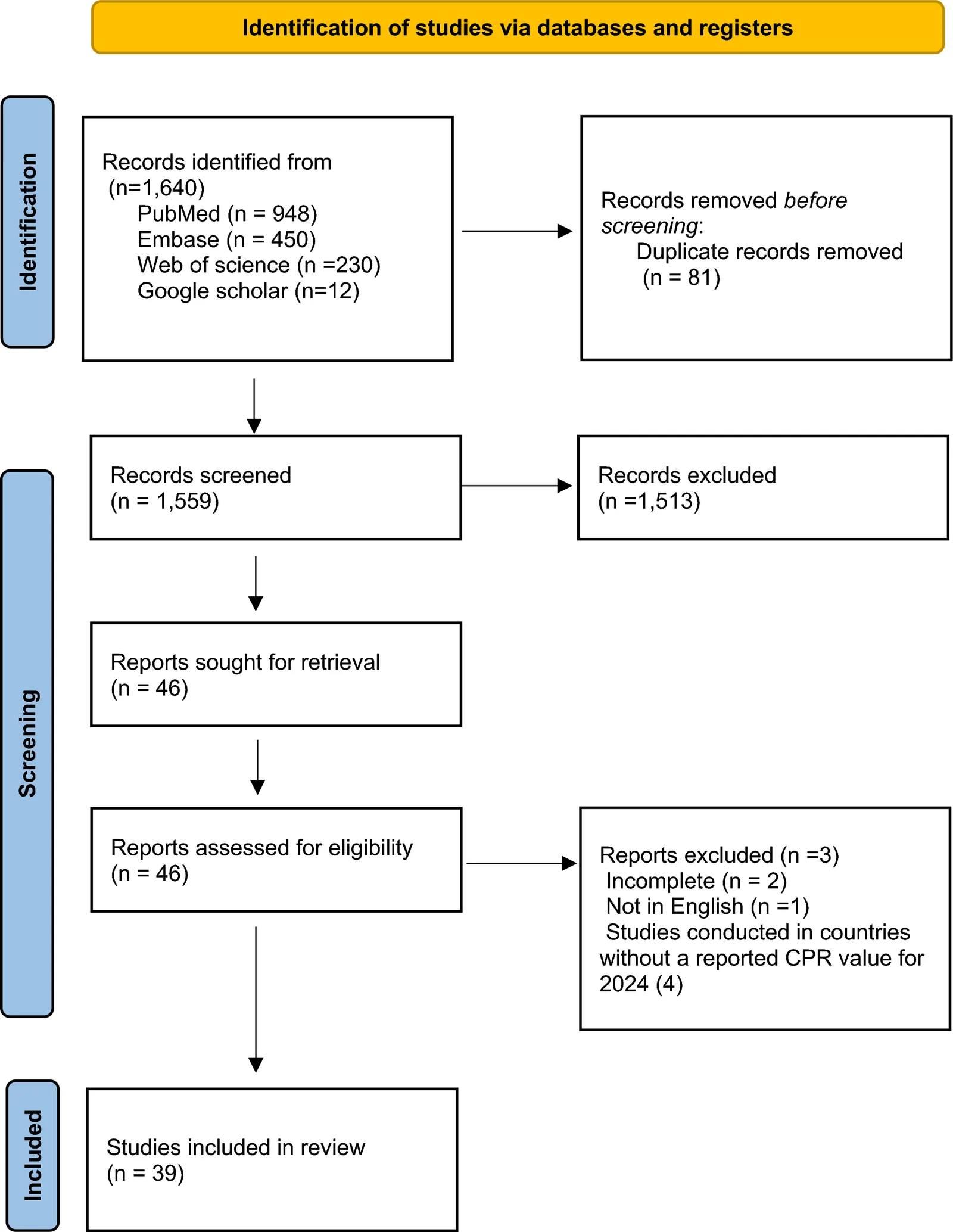
Flow diagram of the study selection process

The search strategy also included citation tracking of relevant articles and a manual search of the reference lists of included studies to identify additional potentially relevant studies. Grey literature sources, such as government reports, conference abstracts, and dissertations, were searched to minimize publication bias. The search was conducted from 15 September to 30 November, 2025. The search strategy used in PubMed is shown in Fig. 1

### Data extraction

Data were extracted using a Microsoft Excel extraction sheet intended for this purpose. The data extraction tools included study characteristics (author, publication year, study design), participant characteristics (demographics, socioeconomic status), intervention details (if applicable), and outcome measures. The data extraction process was conducted independently by two reviewers.

### Eligibility criteria

Quantitative studies, including cross-sectional, cohort, case–control, and randomized controlled trials (RCTs), were eligible for inclusion. Studies focusing on individuals of any age group, gender, or socioeconomic background who have been diagnosed with malaria in SSA were eligible for inclusion. By encompassing a broad population range, the review aimed to capture the diverse experiences and costs incurred by different demographic groups. Studies that assessed direct medical malaria treatment cost incurred by households and the health system were included. Household costs include consultation, diagnosis, antimalarial drugs, and other medications. Health system costs include diagnostics, antimalarial/other drugs, personnel, and hospitalization and etc…

### Study selection

Two independent reviewers screened the identified studies based on predefined inclusion and exclusion criteria. All retrieved records were imported to EndNote version 20 software, and duplicates were removed. All authors screened the titles and abstracts of retrieved studies. Then, the full texts of included studies were reviewed against the eligibility criteria. Disagreements between two screeners were resolved through discussion.

### Quality assessment and risk of bias

The quality assessment was conducted using appropriate tools according to the study design. The Joanna Briggs Institute (JBI) Critical Appraisal Checklist for Cross-Sectional Studies was used to evaluate the quality of the included studies. The checklist includes items assessing sample representativeness, sample size, measurement of exposure, identification and control of confounding factors, and appropriateness of statistical analysis. Each study was evaluated based on the presence or absence of these items.

### Data synthesis

A narrative synthesis was conducted to summarize the findings of the included studies. This study involved a systematic review and synthesis of the characteristics and findings of individual studies, including outpatient and inpatient malaria costs incurred by households and health systems in SSA. Pooled estimates of the mean direct outpatient and inpatient malaria treatment costs borne by households and health systems were also calculated. Random-effects models of the Residual Maximum Likelihood (REML) method was used to calculate summary estimates of household and health system cost, along with their corresponding 95% confidence intervals. Heterogeneity among the studies was assessed using statistical tests (e.g., *I*^*2*^ statistic). STATA version 16 was used for the analysis. To assess potential heterogeneity in the study result, subgroup analyses were conducted based on study population (children under five years versus all age groups, including children, young adults, and older adults) and malaria treatment modality (inpatient versus outpatient care). The results from both the quantitative synthesis were then interpreted together to provide a comprehensive overview of the economic burden of malaria to households and health systems in SSA. To assess publication bias, a funnel plot was generated, and Egger’s regression test was performed, with statistical significance set at p < 0.05.

Both household and health system cost estimates were extracted from the included studies and adjusted to 2024 costs. The local inflation rate method was used to adjust the costs for 2024. The costs from the source literature were collected in United States dollars (USD). The costs were converted to the respective countries' local currencies. To convert the costs to local currencies, the cost is multiplied by the country-specific Purchasing Power Parity (PPP) exchange rate of the origin year. The Purchasing Power Parity (PPP) exchange rate refers to the amount of a country's currency needed to purchase the same quantity of goods and services that can be bought with US$1 in the United States.

The Consumer Price Index (CPI) is used to account for inflation over time and standardize all estimates to 2024 values. The cost adjustments for 2024 were performed using the formula:

$${\text{Cost adjustment 2}}0{24 } = \, \left( {{\mathrm{CPI}}\_{2}0{24 }/{\text{ CPI}}\_{\mathrm{origin}}\_{\mathrm{year}}} \right) \, \times {\text{ Original}}\_{\text{ cost}}$$

After the adjustment, the costs were converted to USD using country-specific PPP exchange rates of 2024. CPI and PPP data were obtained from the World Bank’s World Development Indicators database [13].

## Results

### Search results from review of the studies

We identified 1640 studies through our database search. After excluding duplicate records, we screened 1513 studies that were found irrelevant based on their title and abstracts. Fifty-six studies were reviewed against the eligibility criteria; of these, three articles were excluded (two did not report malaria cost and involved economic evaluation, and one was not published in English. Thirty-Nine of the studies met the inclusion criteria and were included in the final systematic review (Fig. 1).

### Characteristics of the studies in the review

Among included studies, 56% reported household costs, 23% reported health system costs, and 21% of studies reported both the health system and household costs. More than half of the articles' study population across all age groups, and 38% of the studies were focused on children under five. Half of the research articles were published between 2016 and 2023, with six studies appearing between 2022 and 2023 [14–19] (Table 1).

**Table 1:** Characteristics of the included in the systematic review

	Article	Author	Year	Country	Cost perspective	Study population	Study focus [Inpatient (IP) /Outpatient (OP)/Both]	Quality of the study
1	[20]	Batura et al.	2022	Uganda, Mozambique	Health System	Under five	Both	Moderate
2	[21]	Menon et al.	2016	Uganda	Household	Under five	Both	High
3	[22]	Adjagba et al.	2019	Uganda, Burkina Faso Ghana, Nigeria	Household and Health System	All age	Both	Moderate
4	[4]	Alonso et al.	2018	Mozambique	Household and Health System	Under five	Both	High
5	[23]	Castillo-Riquelme et al.	2008	Mozambique, South Africa	Household	All age	OP	Moderate
6	[24]	Watts et al.	2021	Kenya	Household	All age	IP	High
7	[25]	Kodhiambo et al.	2020	Kenya	Household	Under five	IP	Moderate
8	[26]	Ayieko et al.	2009	Kenya	Household and Health System	Under five	IP	Moderate
9	[27]	Maka et al.	2016	Cameroon	Health System	Children age < 15	IP	High
10	[28]	Sieleunou et al.	2015	Cameroon	Household	Under five	OP	Moderate
11	[29]	Mangham-Jefferies et al.	2014	Cameroon	Household and Health System	All age	OP	High
12	[30]	Silumbe et al.	2015	Zambia	Household	All age	Both	Moderate
13	[15]	Cirera et al.	2023	Madagascar, Mozambique, Nigeria	Household	All age	Both	Moderate
14	[31]	Onwujekwe et al.	2013	Nigeria	Health system and Household	All age	Both	High
15	[9]	Ezenduka et al.	2017	Nigeria	Health system	All age	OP	Moderate
16	[16]	Ismail et al.	2023	Nigeria	Household	All age	OP	High
17	[32]	Hennessee et al.	2017	Malawi	Household	All age	IP	High
18	[33]	Ewing et al.	2011	Malawi	Household	Under five	OP	High
19	[34]	Dalaba et al.	2018	Ghana	Household	Under five	OP	High
20	[35]	Tawiah et al.	2016	Ghana	Household	All age	OP	Moderate
21	[36]	Sicuri et al.	2013	Ghana. Tanzania, Kenya	Health system and Household	Under five	Both	Moderate
22	[37]	Mori et al.	2014	Tanzanian	Health system	Under five	Both	High
23	[17]	Masimbi et al.	2022	Rwanda	Health system	All age	Both	High
24	[11]	Hailu et al.	2017	Ethiopia	Household	All age	OP	High
25	[38]	Tefera et al.	2020	Ethiopia	Household	All age	OP	Moderate
26	[6]	Assebe et al.	2021	Ethiopia	Household	All age	OP	High
27	[39]	Devine et al.	2019	Ethiopia	Health system and Household	All age	OP	Moderate
28	[40]	Gunda et al.	2017	Zimbabwe	Household	All age	Both	Moderate
29	[41]	Broekhuizen et al.	2021	Gambia	Household	All age	OP	High
30	[14]	Duval et al.	2022	Gambia, Burkina Faso	Household	Under five	Both	High
31	[42]	Koné et al.	2010	Burkina Faso	Health System	Under five	Both	Moderate
32	[43]	Castellani et al.	2018	Burkina Faso	Household	Under five	IP	Moderate
33	[44]	Maccario et al.	2017	Mali	Health system	Under five	OP	High
34	[45]	Traore et al.	2022	Mali	Health system	Under five	IP	Moderate
35	[46]	Mustafa et al.	2007	Sudan	Household	All age	OP	Moderate
36	[36]	Sicuri et al.	2013	Guinea	Household	All age	Both	High
37	[47]	Romay-Barja et al.	2015	Equatorial Guinea	Health system	All age	OP	High
38	[18]	Sossou et al.	2022	Benin	Household	All age	Both	Moderate
39	[19]	Hezagira et al.	2022	Burundi	Health system and Household	Under five	Both	Moderate

### Small study effect test (assessment of publication bias)

**Fig. 2:**
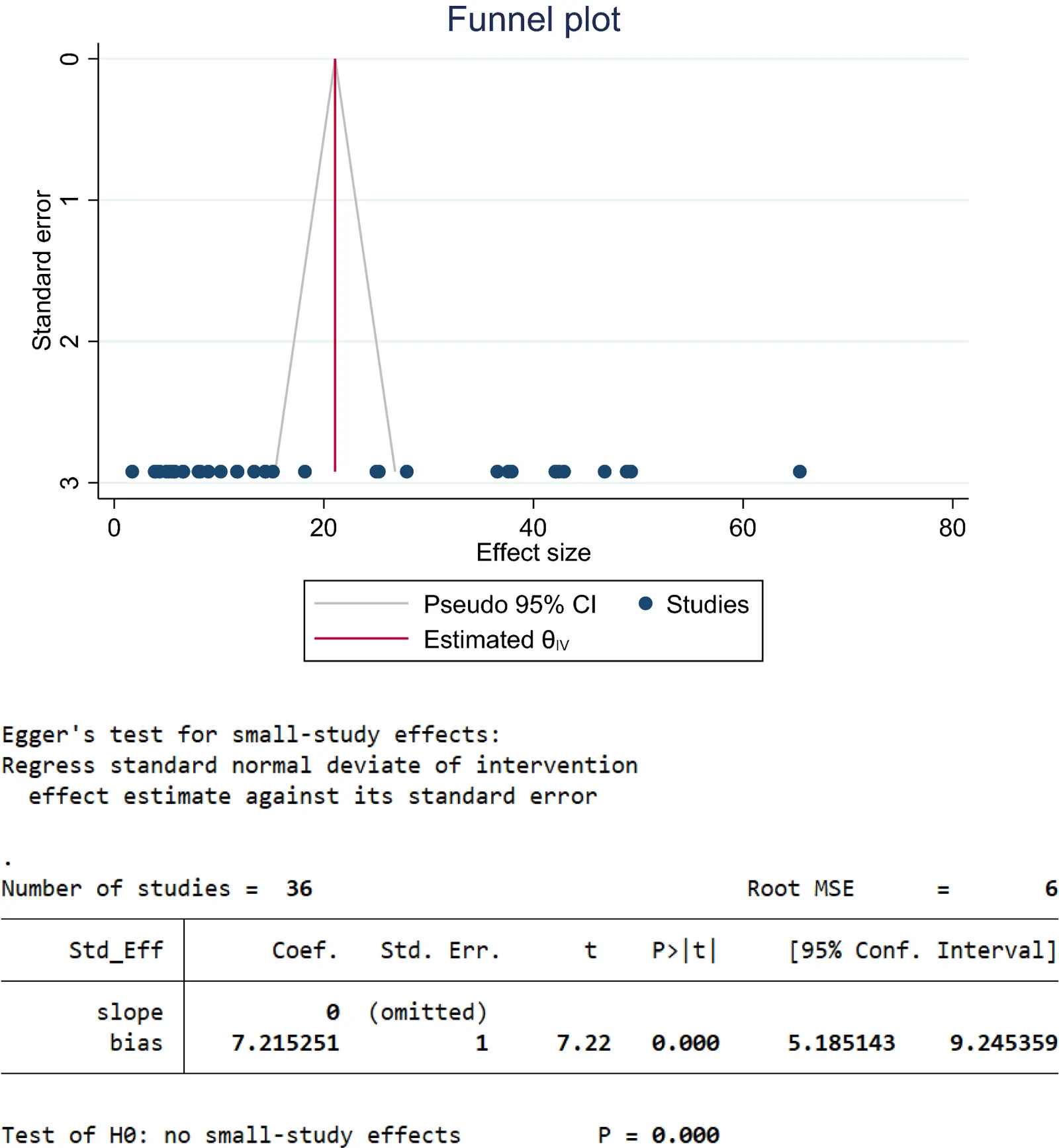
Egger’s test plot for the outpatient cost studies included in the systematic review assessing the pooled estimate of household and health system cost in SSA

**Fig. 3:**
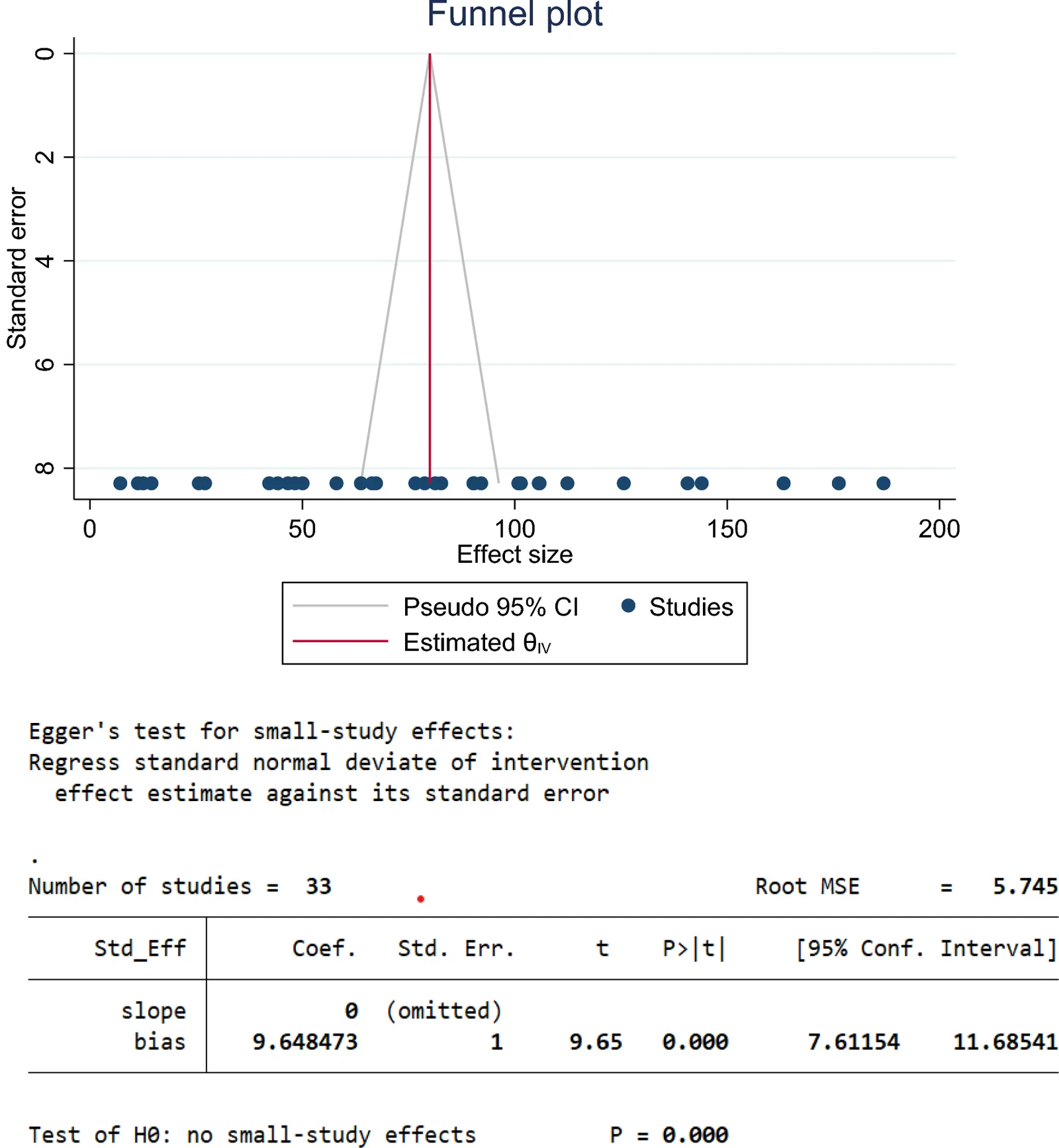
Egger’s test plot for the inpatient cost studies included in the systematic review assessing the pooled estimate of household and health system cost in SSA

Publication bias or small study effect was evaluated using both a funnel plot and Egger’s regression test. The studies were categorized as inpatient and outpatient because the Egger’s test is quite sensitive to heterogeneity. The funnel plots Figs. 2 and 3 demonstrated a relatively asymmetric distribution of effect sizes around the pooled estimate, suggesting the presence of publication bias. In addition, the Egger’s tests showed statistical evidence of small study effects, with a p-value of < 0.001. The findings indicated that the review results are affected by potential bias arising from smaller studies or selective publication.

### Household malaria cost

The pooled household malaria treatment costs were analysed for both outpatients and inpatients in SSA. The pooled outpatient household malaria cost (i.e., uncomplicated episode of malaria) was $12.55 (95% CI $9.58, $16.45, *I*^2^ = 96.29%, P < 0.01). Moreover, subgroup analysis indicated that the pooled outpatient estimated cost for children under five was $9.54 (95% CI $7.23, $12.59, *I*^2^ = 70.98%, P < 0.01) and the outpatient cost among all age groups was $16.29 (95% CI $11.09, $23.91, *I*^2^ = 97.98%, P < 0.01).

**Fig. 4:**
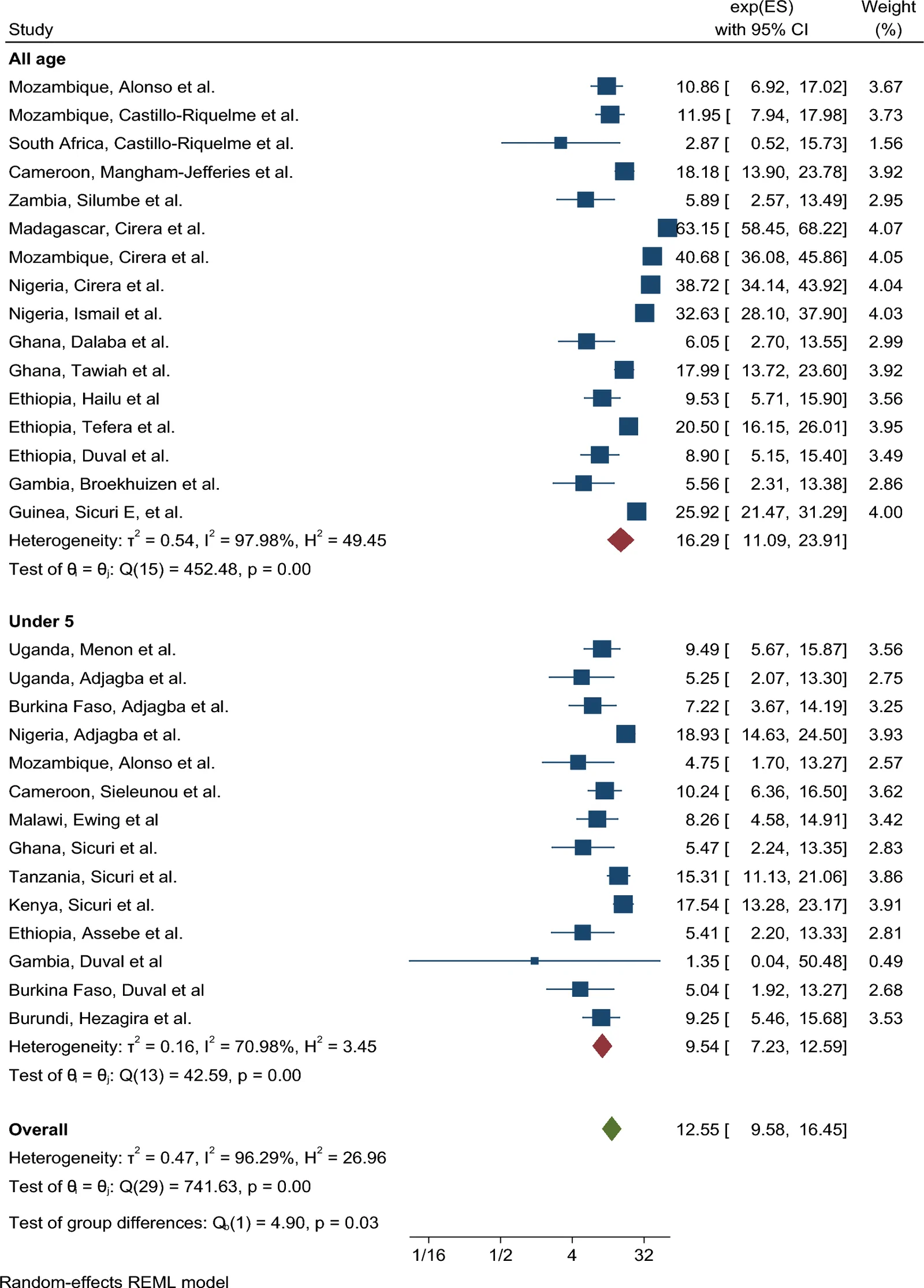
Forest plot showing the pooled household outpatient malaria cost Sub-Saharan Africa counties

The estimated household outpatient cost in the Gambia [41] was $1.35 (95% CI $0.04, $50.48), and the 2024 GDP per capita was $871. In Madagascar [15], the estimated cost was $63.15 (95% CI $58.45, $68.22), where GDP per capita was $545 ( Fig. 4).

**Fig. 5:**
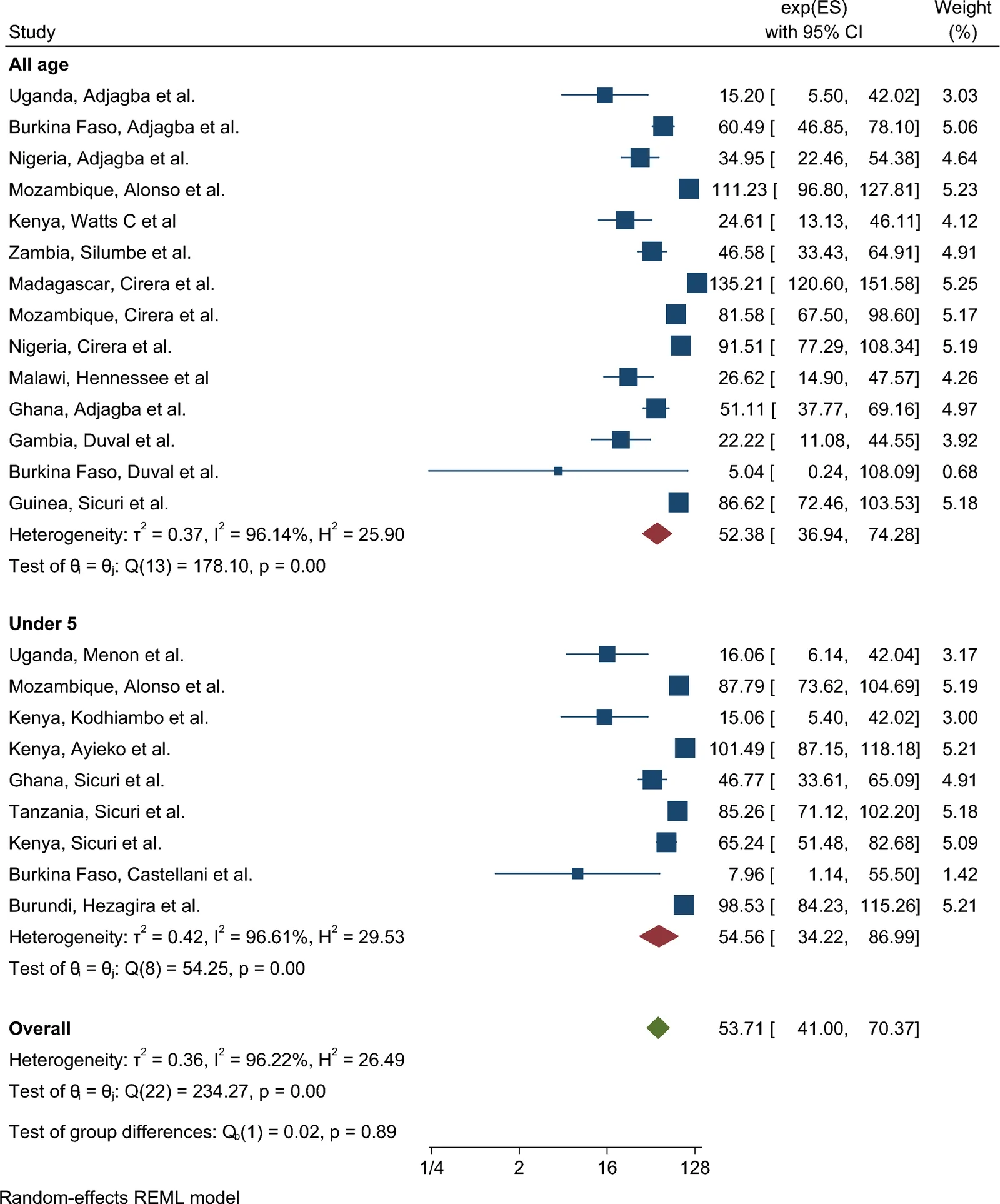
Forest plot showing the pooled household inpatient malaria cost in Sub-Saharan Africa counties

The pooled household’s estimated inpatient cost was $53.71 (95% CI $41.00, $70.31, *I*^2^ = 96.22%, P < 0.01). According to subgroup analysis, the inpatient cost among under five children was $54.56 (95% CI $34.22, $86.99, *I*^2^ = 96.61%, P < 0.01), compared to $52.38 (95% CI $36.94, $74.28, *I*^2^ = 96.14%, P < 0.01) across all age groups. In Madagascar [15], the estimated cost was $135 (95% CI $120.60, 151.58), where the 2024 GDP per capita was $545. The estimated cost in Mozambique [4] was $111.23 (95% CI $96.80, $127.81), while the 2024 GDP per capita was $656 ( Fig. 5).

### Household malaria treatment cost by socioeconomic status

Household malaria treatment costs differ depending on households’ socioeconomic status in SSA. Households in the poorest quantile face the highest costs for malaria treatment compared to the richest quintile, scaled to their economic status [11, 17, 23, 32, 34, 35]. For instance, in Ethiopia, 40% of households in the poor quintile experience catastrophic health expenditure, imposing the greatest financial burden on their income [6, 38]. This highlights the disproportionate economic impact on low-income households in the region.

### Health system malaria cost

**Fig. 6:**
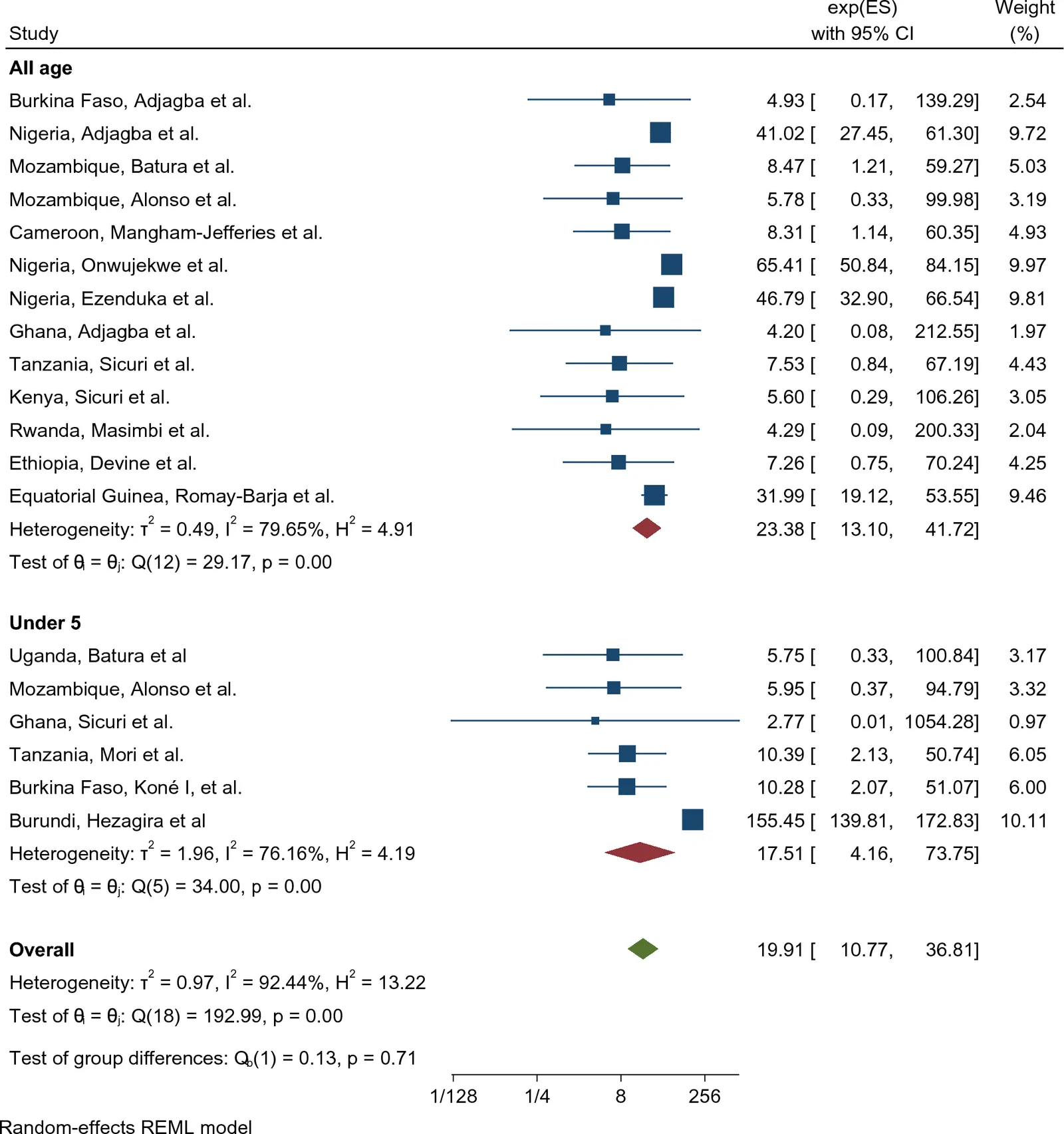
Forest plot showing the pooled health system outpatient malaria cost in Saharan Africa countries

The pooled outpatient health system cost for malaria treatment was $19.91 (95% CI $10.77, $36.81, *I*^*2*^ = 92.44%, P < 0.01). The subgroup analysis indicated that the pooled outpatient health system cost for children under five was $17.51 (95% CI $4.16, $73.75, *I*^*2*^ = 76.16%, P < 0.01), and $23.38 (95% CI $13.10, $41.72,* I*^*2*^ = 79.65%, P < 0.01) across all age groups in the region. The estimated cost in Burundi [19] was $155.45 (95% CI $139.81, $172.83), while the 2024 GDP per capita was $219. In Ghana [36], the estimated cost was $2.77 (95% CI $ 0.01, $1054.28), while the 2024 GDP per capita was $2390 (Fig. 6).

**Fig. 7:**
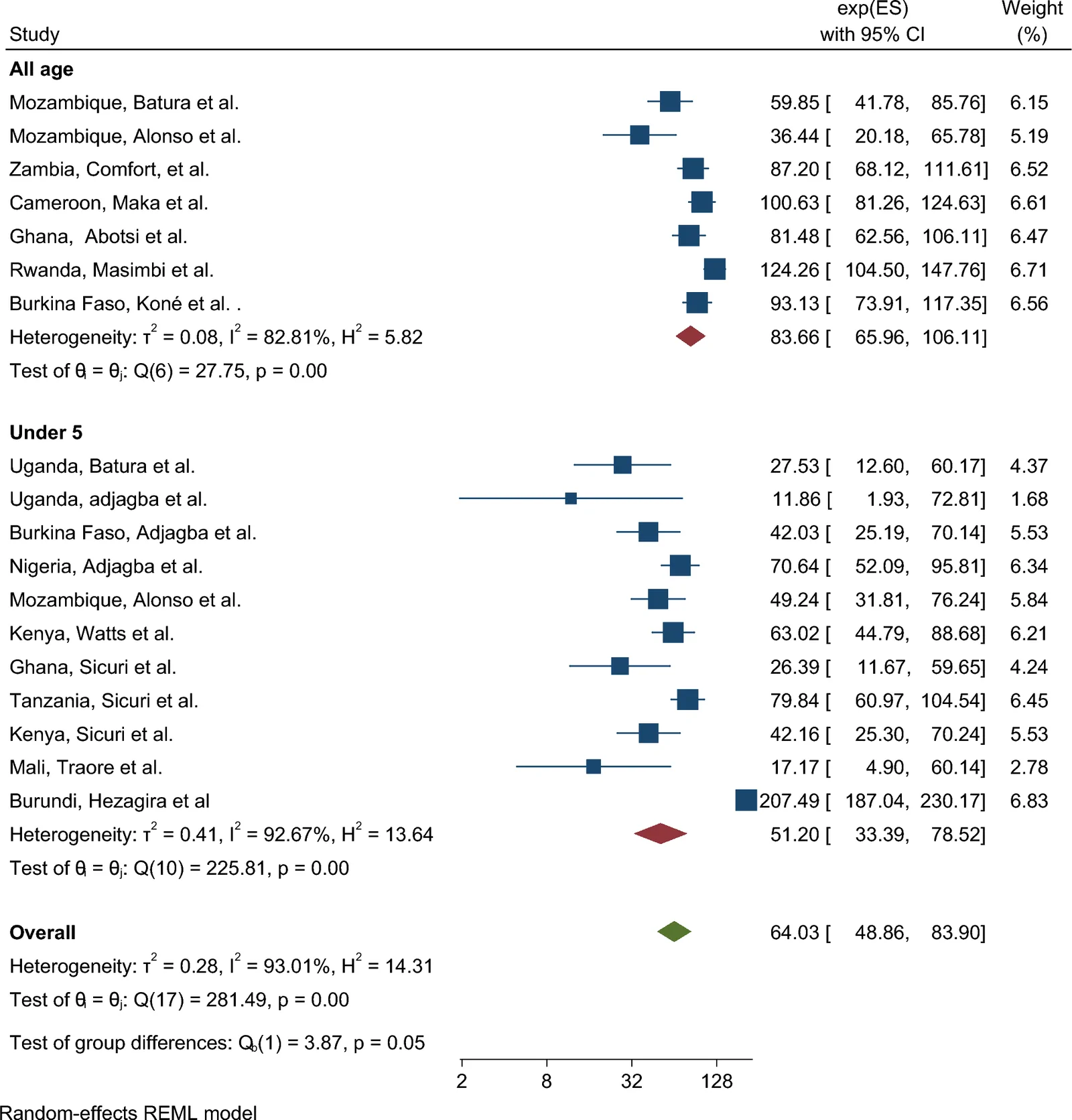
Forest plot showing the pooled health system inpatient malaria cost in Saharan Africa countries

The pooled estimated health system inpatient cost of severe malaria treatment was $64.03 (95% CI $48.86, $83.90,* I*^*2*^ = 93.01%, P < 0.01). The inpatient cost for children under five was $51.20 (95% CI $33.39, $78.52,* I*^*2*^ = 92.67%, P < 0.01), and the cost across all age groups was $83.66 (95% CI $65.96, $106.11,* I*^*2*^ = 82.81%, P < 0.01). Comparing the cost across countries, Burundi [19] had an estimated cost of $207.49 (95% CI $187.04, $230.17), which was close to the country’s 2024 GDP per capita of $219. In Kenya [24], the estimated cost was $63.02 (95% CI $44.79, $88.68), and the 2024 GDP per capita was $ 2132 ( Fig. 7).

## Discussion

This review synthesizes evidence from 39 studies published between 2008 and 2023 on malaria costs in SSA. The pooled household malaria treatment cost for outpatient (i.e., uncomplicated) per case was $12.55 (95% CI $9.58, $16.45) and $53.71 (95% CI $41.00, $70.37) per inpatient (severe malaria) episode. Regarding health system costs, the pooled outpatient malaria treatment cost was $19.91 (95% CI $10.77, $36.81), while the inpatient treatment cost was $64.03 (95% CI $48.86, $83.90). The pooled household inpatient cost was approximately four times the household outpatient cost in the region. The observed variation in cost was due to the high expenses of medication, laboratory tests, and hospitalization for inpatient costs compared to outpatient costs [10].

Subgroup analysis by study population revealed variations in the pooled outpatient household costs among population subgroups. The pooled household outpatient malaria treatment cost for under-five children was $9.54 (95% CI $7.23, $12.59) compared to the cost across all age groups, which was $16.29 (95% CI $11.09, $23.91). This might be due to smaller drug dosages, free or subsidized malaria diagnosis, and treatment service initiatives targeted at children under five in SSA [48–50].

There was inequality in households’ malaria treatment cost across socioeconomic status in SSA. Households in the poorer quintile faced higher costs than those in the richer quintile, leading poor households to suffer catastrophic health expenditures related to malaria treatment costs [51]. Low economic conditions and inadequate funding for malaria treatment contributed to poor households spending most of their income on malaria treatment in SSA [52].

Both household and health system malaria costs varied across the countries involved in the studies. The difference might be due to varying costing methods or malaria treatment guidelines used across the countries. In addition, factors such as limited health insurance coverage [34, 53], distance from healthcare facilities [11, 32, 33], type of facility providing treatment (e.g., health centres vs. primary hospitals) [11, 32] might contributed to the variations in malaria treatment costs among households and health systems across countries. Moreover, the presence of comorbidities and inpatient hospital stay were also drivers for the cost variation [24, 26, 36].

In addition, low-income countries, such as Burundi and Madagascar, had a higher burden of the malaria treatment costs compared to lower- and middle-income countries, such as Ghana and Kenya, in the region. The disparities in economic strength among the countries likely contribute to the variation in health infrastructure and funding, which in turn explains the difference in malaria treatment costs across countries [33, 35, 54].

Moreover, there was considerable heterogeneity in estimates among the studies and subgroups. These differences may be attributable to the varied settings from which costing data were collected and the types of health facilities where malaria care was sought. Similarly, the cost of treatment may vary within the countries based on the treatment guideline and availability of resources in different settings, contributing to some heterogeneity in reported costs.

We acknowledge several limitations of this study. First, for the methodological limitations, there was substantial heterogeneity among the individual studies regarding study setting and facility type. We did not perform the analysis based on cost classifications such as direct and indirect costs, limiting our understanding of the influence of different cost types. We, thus, could not account for all of these factors simultaneously due to data limitations. Therefore, our estimates of malaria management cost should be interpreted as a crude median cost. Second, due to data gaps, we could not estimate the cost of malaria management according to aetiology, particularly for falciparum or vivax.

## Conclusions

Malaria imposed a substantial and unequal economic burden to the households and health systems cross SSA. Low income households disproportionately impacted by malaria treatment cost. Malaria also had a significant economic strain on health systems in low-income countries in the region. Strengthening health system capacity and improving access to affordable malaria treatment are essential to reduce this burden and support more efficient allocation of resources in the region.

## Supplementary Information

Additional file 1.

## Data Availability

No datasets were generated or analysed during the current study.
